# Tailoring the electrochemical properties of halogen-free electrolytes by dimethyl sulfoxide additives for magnesium battery applications

**DOI:** 10.1039/d6ra03264h

**Published:** 2026-07-03

**Authors:** Awad M. Bakry, Shuayl Alotaibi, L. S. El-Sherif, Safwat Hassaballa, Shrouk Behalo, Eslam Sheha

**Affiliations:** a Department of physics, College of Science and Humanities in Al-Kharj, Prince Sattam bin Abdulaziz University Al-Kharj 11942 Saudi Arabia; b Physics Department, Faculty of Science, Islamic University of Madinah Almadinah Al-Munawarah 42351 Saudi Arabia; c Physics Department, Faculty of Science, Benha University 13518 Benha Egypt islam.shihah@fsc.bu.edu.eg

## Abstract

Halogen-free electrolytes (HFEs) are promising for rechargeable magnesium batteries, but their application is limited by moderate ionic conductivity and interfacial instability. In this work, dimethyl sulfoxide (DMSO) was introduced as a functional additive to tailor the physicochemical and electrochemical properties of a Mg(NO_3_)_2_-based HFE system. FTIR and UV-vis analyses suggested that DMSO modifies the solvation structure through strong Mg^2+^–O coordination, leading to enhanced polarizability and reduced optical band gap. Electrochemical results showed improved ionic conductivity (up to 1.7 × 10^−3^ S cm^−1^), lower activation energy (∼0.10 eV), higher Mg^2+^ transference number (∼0.82), improved apparent oxidative stability (∼3.7 V), and improved Mg stripping/plating behavior with reduced overpotential. Mg–S full cells with sulfur/graphene/BaTiO_3_ cathodes exhibited enhanced discharge/charge capacities (1732/1176 mAh g^−1^) and improved cycling performance. XRD, SEM/EDS, impedance, and diffusion analyses further suggested enhanced Mg^2+^ transport and stabilization of the Mg/electrolyte interface. Overall, DMSO-induced solvation engineering provides an effective strategy for improving halogen-free electrolytes and magnesium battery performance.

## Abstract 2

Halogen-free electrolytes (HFEs) are promising for rechargeable magnesium batteries, but their application is limited by moderate ionic conductivity and interfacial instability.

## Graphical abstract

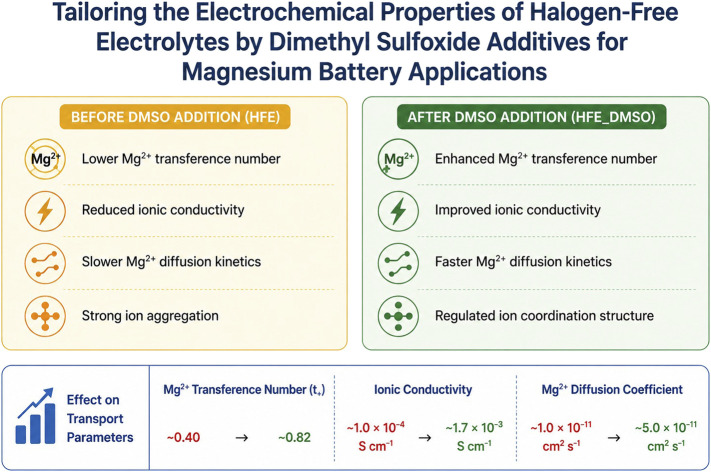

## Introduction

Achieving high-efficiency, safe, and sustainable electrochemical energy storage remains a central challenge in modern battery research. While lithium-ion batteries dominate current applications, concerns related to resource scarcity, cost, and safety have driven increasing interest toward alternative multivalent systems.^1^ Among these, rechargeable magnesium batteries have emerged as promising candidates due to their high volumetric capacity, natural abundance, dendrite-free deposition behavior, and improved safety profile.^2^ However, despite these advantages, their practical realization remains limited by fundamental challenges associated with electrolyte compatibility and sluggish Mg^2+^ transport.^3^ In our previous work,^4^ we introduced a simple halogen-free electrolyte (HFE) based on Mg(NO_3_)_2_ dissolved in an ACN/G4 cosolvent system, demonstrating that such electrolytes can enable reversible Mg plating/stripping with relatively high ionic conductivity (∼10^−4^ S cm^−1^) and improved anodic stability. Moreover, the electrolyte supported dendrite-free Mg deposition and enabled the operation of Mg–S full cells with initial discharge capacities approaching 1200 mAh g^−1^. Despite these promising results, several key limitations remained, including moderate coulombic efficiency, interfacial instability, and limited long-term cycling performance. Importantly, our previous study explicitly highlighted that further optimization of solvation structure and the introduction of high donor-number solvents such as dimethyl sulfoxide (DMSO) could provide an effective pathway to enhance electrolyte performance.^4^ From a broader research perspective, the electrolyte remains the most critical bottleneck in magnesium battery systems. Conventional chloride- or Lewis-acid-based electrolytes, although effective in enabling Mg stripping/plating, suffer from corrosivity, air sensitivity, and incompatibility with high-voltage cathodes. Additionally, the strong polarizing nature of Mg^2+^ leads to ion pairing and the formation of passivating interphases at the Mg surface, which significantly increases overpotential and suppresses reversible electrochemistry.^5,6^ Recent advances in electrolyte design emphasize a new paradigm based on solvation-structure engineering and functional additives. It has been demonstrated that introducing suitable additives can significantly enhance salt dissociation, regulate ion-solvent interactions, improve Mg plating/stripping efficiency, and widen the electrochemical stability window.^7^ For example, methoxyethyl-amine chelants have been shown to enhance interfacial charge transfer and suppress side reactions *via* solvation sheath reorganization, enabling stable cycling with energy densities up to 412 and 471 Wh kg^−1^, respectively. This highlights a versatile electrolyte design strategy for divalent metal batteries.^7^ Similarly, multifunctional additives in Mg-borohydride systems have been shown to dramatically increase ionic conductivity, improve coulombic efficiency (>99%), and enable stable Mg–S battery operation by forming new active ionic species and tuning the electrolyte structure.^6^ Within this framework, dimethyl sulfoxide (DMSO) represents a particularly promising additive due to its unique physicochemical properties. DMSO is a highly polar aprotic solvent with a large dielectric constant (∼46), strong coordinating ability *via* the S=O group, wide electrochemical window, and low toxicity.^8^ Previous studies, including our own work on MgBr_2_/DMSO systems, have demonstrated that DMSO can effectively support Mg^2+^ transport and enhance ionic conductivity in non-aqueous electrolytes.^9^ More importantly, recent investigations on halogen-free Mg electrolytes revealed that DMSO plays a critical role in modifying the Mg/electrolyte interface, reducing polarization, increasing Mg^2+^ transference number, and stabilizing long-term stripping/plating behavior, thereby directly addressing the key limitations observed in earlier HFE systems.^10^ In addition, insights from recent electrolyte-additive studies in lithium systems further reinforce this mechanism. The incorporation of DMSO has been shown to enhance ion dissociation, increase ionic conductivity, and improve electrochemical stability by strengthening cation–solvent interactions and modifying the solvation shell. These findings provide a strong conceptual basis for extending DMSO-based solvation engineering to magnesium electrolytes.^11^ Motivated by these findings and building directly on our previous HFE platform, the present work aims to develop an improved halogen-free electrolyte system through the controlled incorporation of DMSO as a functional additive. The central hypothesis is that DMSO can regulate the solvation structure of Mg^2+^ ions, reduce ion pairing, enhance ionic mobility, and stabilize the Mg/electrolyte interface, thereby overcoming the limitations observed in earlier Mg(NO_3_)_2_-based electrolytes. In parallel, the study integrates a sulfur-based composite cathode with functional additives to further enhance electrochemical performance. The sulfur/graphene/BaTiO_3_ composite cathode was employed to enhance electronic conductivity and interfacial stability during Mg^2+^ insertion/extraction. Graphene improves electronic transport within the cathode matrix, while BaTiO_3_ may suppress polysulfide dissolution through interfacial adsorption and polarization effects, thereby reducing active material loss during cycling. Nevertheless, the primary focus of this work remains the development of the DMSO-modified halogen-free electrolyte, whereas the composite cathode serves as a compatible model system for evaluating electrolyte performance. ^12,13^ This dual-engineering strategy-combining electrolyte modification and cathode optimization-is expected to deliver synergistic improvements in Mg^2+^ transport, reaction kinetics, and overall cell stability. The electrochemical properties of the developed electrolytes are systematically evaluated using electrochemical impedance spectroscopy (EIS), Mg stripping/plating measurements, cyclic voltammetry (CV), and linear sweep voltammetry (LSV). Furthermore, full Mg-based cells are assembled to assess practical performance, while structural and morphological analyses (XRD, SEM, and EDS) are conducted to elucidate the underlying magnesium storage mechanisms and interfacial evolution. It is anticipated that the incorporation of DMSO will lead to enhanced ionic conductivity, higher Mg^2+^ transference number, reduced overpotential, and improved cycling stability. More broadly, this work aims to establish a clear structure–property relationship between additive-induced solvation tuning and electrochemical performance, providing a rational pathway for designing next-generation halogen-free electrolytes for magnesium batteries.

## Experimental technique

Magnesium nitrate hexahydrate, Mg(NO_3_)_2_·6H_2_O (Alfa Aesar, 98%), was dehydrated in a vacuum oven at 80 °C for 48 h to minimize residual moisture. In parallel, molecular sieves were activated by microwave heating for 10 min prior to use. The electrolyte was prepared by dissolving 3.39 g of pretreated magnesium salt in a mixed solvent consisting of 12.88 mL acetonitrile (ACN) and 3.10 mL tetraethylene glycol dimethyl ether (G4) under an argon atmosphere. Activated molecular sieves were then introduced into the solution to further remove trace amounts of water, and the mixture was allowed to stand for 24 h to ensure effective dehydration. The solution was subsequently stirred at 200 rpm until a clear and homogeneous halogen-free electrolyte (HFE) was obtained. Dimethyl sulfoxide (DMSO) was then added as a functional additive in varying amounts to tailor the electrolyte properties, and the resulting systems are denoted as HFE@x µL DMSO, where *x* = 0, 100, 200, 300, and 400 µL. Mg(NO_3_)_2_·6H_2_O was vacuum-dried at 80 °C for 48 h, and activated molecular sieves were added to further reduce residual moisture. The electrolyte concentration was approximately ∼1 M. Although trace water may still be present, the adopted dehydration procedure provided sufficiently low moisture content for stable electrochemical performance. The composite cathode material was prepared by mixing sulfur (S, Alfa Aesar, 99%), graphene, and barium titanate (BTO, Alfa Aesar, 99%) in a mass ratio of 75 : 20 : 5 wt%, followed by thorough grinding to ensure uniform dispersion. For electrode fabrication, the active composite (75 wt%) was blended with Super P conductive carbon (10 wt%) and polyvinylidene fluoride (PVDF, 15 wt%) as a binder. The mixture was dispersed in *N*-methyl-2-pyrrolidone (NMP) and stirred continuously for 24 h to form a homogeneous slurry. The slurry was then coated onto aluminum foil using a Hosen M20 mini-coater with a controlled thickness of approximately 100 µm. The coated electrodes were dried at 100 °C for 2 h to remove residual solvent, followed by further drying at 65 °C under vacuum, and then stored in a vacuum oven prior to cell assembly, the active sulfur mass loadings were 0.45 mg cm^−2^. Mg–S full cells were assembled in CR2032 coin cells using Mg foil as the anode (0.1 mm thickness, 16 mm diameter), a glass microfiber separator, the developed halogen-free electrolyte, and a sulfur/graphene/BaTiO_3_ composite cathode coated on Al foil. For post-cycling analysis, symmetric Mg//Mg cells were subjected to galvanostatic cycling for 30 cycles, after which the magnesium anodes were retrieved for structural and morphological characterization. For cathode analysis, cells were disassembled at defined states, namely after discharge to 0.25 V and after subsequent charging to 2.5 V, and the recovered electrodes were collected for further investigation. Galvanostatic charge–discharge measurements were performed using a NEWARE BTS4000 battery testing system at 25 °C with a current density of 20 µA cm^−2^ within a voltage window of 0.25–2.5 V. Cyclic voltammetry (CV) and electrochemical impedance spectroscopy (EIS) measurements were conducted using CHI6004E and CHI6005E electrochemical workstations with EIS measurements carried out over a frequency range from 1 MHz to 1 Hz at room temperature. The electrochemical stability window of the electrolyte was evaluated by linear sweep voltammetry (LSV) using Mg//electrolyte//stainless steel cells at a scan rate of 0.02 V s^−1^ over a voltage range of −1.0 to 5.0 V. The magnesium-ion transference number was determined using a combination of AC impedance and DC polarization measurements in symmetric Mg//Mg cells. To ensure the reproducibility of the electrochemical results, all measurements were repeated using at least three independently assembled coin cells under identical experimental conditions, and the reported electrochemical performance values represent the average results obtained from these measurements.^14^ X-ray diffraction (XRD) patterns were recorded using a Rigaku MiniFlex 600 diffractometer with Cu Kα radiation (*λ* = 1.5406 Å), employing a step size of 0.02° and a scan time of 3 s per step. Surface morphology and elemental composition were analyzed using a scanning electron microscope (SEM, JCM-7000 NeoScope, JEOL) equipped with an energy-dispersive X-ray spectroscopy (EDS) detector. Fourier-transform infrared (FTIR) spectra were collected using an a Bruker ALPHA II FTIR spectrometer, while UV-vis absorption spectra were obtained using Edinburgh DS5 dual-beam spectrophotometer. The impedance data were fitted and analyzed using ZView software.

## Results and discussion

**Fig. 1:**
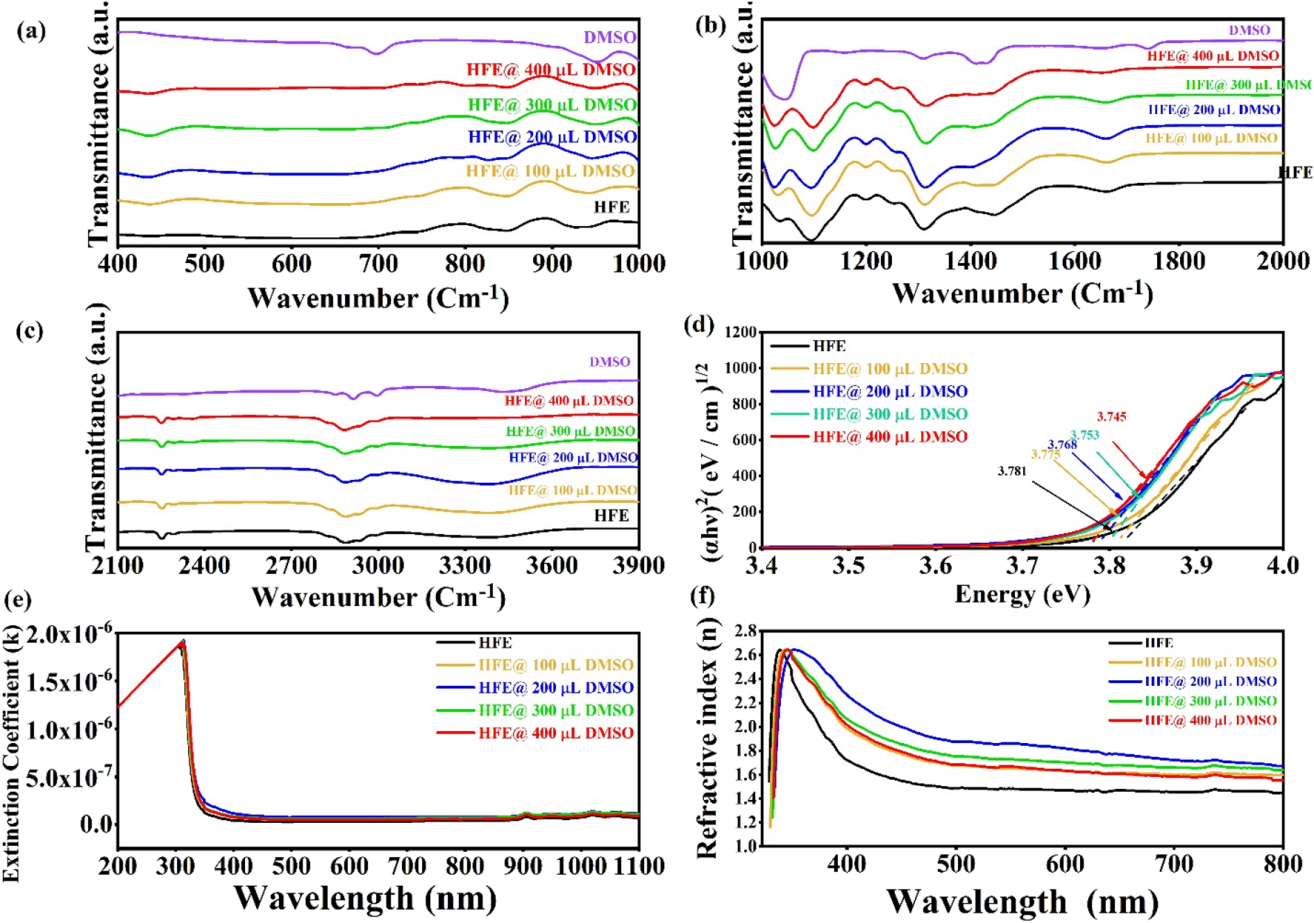
(a–c) FTIR spectra of HFE@x µL DMSO (*x* = 0–400). (d) Tauc plot. (e) Extinction coefficient *vs.* wavelength. (f) Refractive index *vs.* wavelength.

Fig. 1(a–c) presents the FTIR spectra of the halogen-free electrolyte (HFE) and its modification with different concentrations of dimethyl sulfoxide (DMSO), clearly illustrating the effect of DMSO incorporation on the vibrational structure of the system. In the region of 1000–1200 cm^−1^, a characteristic band located at ∼1027 cm^−1^ is assigned to the S=O stretching vibration of DMSO.^15^ Upon increasing the DMSO content to 300 and 400 µL, this band exhibits a slight shift toward lower wavenumbers (∼1024 cm^−1^), indicating a weakening of the S=O bond. This red shift is attributed to coordination interactions between the oxygen atom of DMSO and Mg^2+^ ions, which reduces the bond force constant and suggests the formation of a modified solvation structure rather than a simple dilution effect. In the same spectral region, the band observed at ∼1091 cm^−1^ corresponds to the C–O–C stretching vibration of the ether groups in the G4 solvent.^16^ With increasing DMSO concentration, this band shifts slightly to higher wavenumbers (∼1096–1098 cm^−1^), suggesting a strengthening of the C–O–C bonding environment due to changes in intermolecular interactions and redistribution of Mg^2+^ coordination between G4 and DMSO molecules. The band at ∼1310 cm^−1^, attributed to C–H bending vibrations, also shows a minor blue shift to ∼1313 cm^−1^ at higher DMSO concentrations, reflecting increased bond stiffness and enhanced dipole–dipole interactions within the modified electrolyte system.^17^ In the higher wavenumber region, several characteristic bands are identified, including a band at ∼1660 cm^−1^ associated with coordinated nitrate or weak C=O-type vibrations, a distinct band at ∼2250 cm^−1^ corresponding to the C≡N stretching vibration of acetonitrile (ACN), bands in the range of ∼2884–3000 cm^−1^ assigned to C–H stretching modes, and a broad band around ∼3400 cm^−1^ attributed to O–H stretching from trace moisture or hydrogen-bonded species.^18^ The slight shifts and intensity variations observed in these bands with increasing DMSO content indicate modifications in hydrogen bonding and solvent-ion interactions, further suggesting the restructuring of the electrolyte environment. The observed shifts in vibrational frequencies can be interpreted based on Hooke's law, where the vibrational frequency depends on the bond force constant; a shift toward lower wavenumbers (red shift) reflects bond weakening, as observed for the S=O group due to Mg^2+^ coordination, whereas a shift toward higher wavenumbers (blue shift) indicates bond strengthening or reduced intermolecular coupling, as observed for the C–O–C and C–H vibrations. The FTIR and optical measurements were reproducible within the experimental uncertainty, and the observed variations showed systematic evolution with increasing DMSO concentration. Although some spectral shifts are relatively small, their consistent trend supports gradual modification of the Mg^2+^ solvation environment. The band near ∼1027 cm^−1^ is mainly attributed to the S=O stretching vibration of coordinated DMSO; however, partial overlap with G4 ether-related and nitrate-associated vibrations cannot be completely excluded. The progressive shift and intensity evolution with increasing DMSO content nevertheless support DMSO-induced solvation modification. Although some optical parameters showed more pronounced variation at intermediate DMSO concentrations, the HFE@400 µL DMSO composition exhibited the best overall electrochemical performance and was therefore selected for detailed electrochemical investigation.^19^ The optical band gap of the prepared electrolytes was evaluated using the Tauc relation,^20^ expressed as:

1 (*αhν*)^*n*^ = *K*(*hν* − *E*_g_)

where *α* is the absorption coefficient, *h* is Planck's constant, *ν* is the photon frequency, *K* is a proportionality constant, and *E*_g_is the optical band gap. The exponent *n* depends on the nature of the electronic transition; *n* = 2corresponds to a direct allowed transition, while 
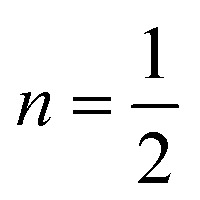
 corresponds to an indirect transition. In the present work, the plots of (*αhν*)^2^*versus* photon energy (Fig. 1(d)) indicate that the optical transitions are predominantly direct allowed transitions. The band gap values were determined by extrapolating the linear region of each curve to the energy axis. The extracted values show a gradual decrease in band gap from approximately 3.781 eV (HFE) to 3.745 eV (HFE@400 µL DMSO). This systematic reduction in *E*_g_ with increasing DMSO concentration suggests the formation of additional localized electronic states within the band structure. These states are likely associated with strong coordination interactions between Mg^2+^ ions and the oxygen atom in the S=O group of DMSO, which modifies the solvation environment and electronic structure of the electrolyte. Consequently, the narrowing of the band gap indicates enhanced electronic polarizability and improved charge-transfer characteristics, which can positively influence ionic transport behavior. The extinction coefficient *k*, which describes the attenuation of electromagnetic waves due to absorption and scattering, was calculated using:^21^2
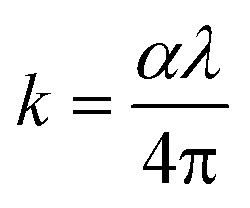
where *λ* is the wavelength of incident light. As shown in Fig. 1(e), all samples exhibit a strong absorption edge in the UV region (∼200–320 nm), characterized by a sharp increase in *k*. Beyond this region, the extinction coefficient decreases rapidly and approaches near-constant values in the visible and near-infrared regions. The incorporation of DMSO leads to a slight increase in *k* in the UV region, indicating enhanced photon–matter interaction due to increased electronic polarization and improved dipole activity associated with the S=O functional group. In contrast, the relatively low and stable values of *k* in the visible region suggest that the electrolyte systems remain optically transparent, which is advantageous for minimizing parasitic optical losses and indicates the absence of significant defect-induced absorption in this spectral range. The refractive index *n*, which reflects the propagation of electromagnetic waves within the medium, was evaluated using its relation to the optical constants:^22^3
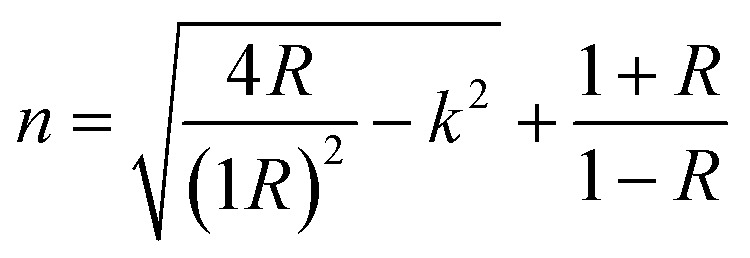
where *R* is the reflectance and *k* is the extinction coefficient. The relation *A* + *R* + *T* = 1 (where *A*, *R*, and *T* are absorption, reflectance, and transmittance, respectively) was used to ensure consistency of optical parameters. As illustrated in Fig. 1(f), the refractive index exhibits a maximum in the UV region (∼330–350 nm) followed by a gradual decrease toward longer wavelengths. The addition of DMSO results in a notable increase in the refractive index across the entire spectral range, with the highest values observed for HFE@200 µL DMSO. This behavior indicates that DMSO enhances the optical density and polarizability of the electrolyte, leading to stronger interaction with the electromagnetic field and reduced phase velocity of light within the medium. The subsequent decrease in *n* at higher wavelengths reflects normal dispersion behavior, where photon energy becomes insufficient to excite electronic transitions, resulting in improved transparency and faster wave propagation.

**Fig. 2:**
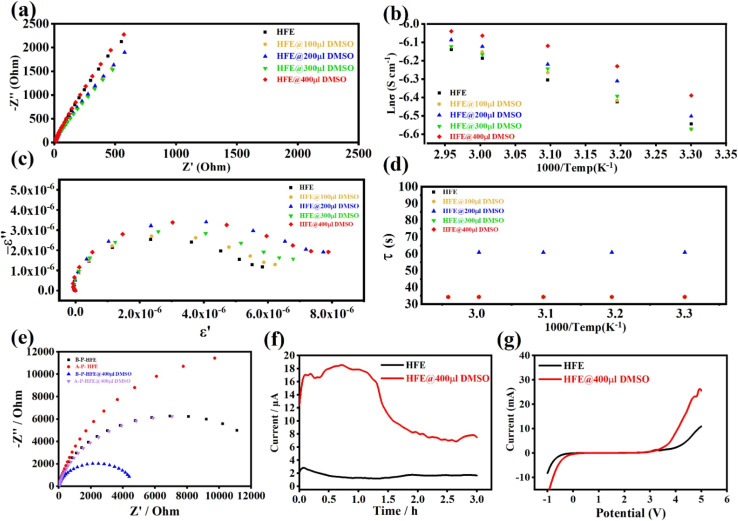
(a) Nyquist plots of HFE@x µL DMSO. (b) ln(σ) *vs.* 1000/T. (c) *ε*″ *vs. ε*′. (d) *τ vs.* temperature. (e and f) Polarization and impedance. (g) LSV curves.

Fig. 2(a) presents the Nyquist plots of HFE@x µL DMSO electrolytes (*x* = 0, 100, 200, 300, and 400). All spectra exhibit a depressed semicircle in the high-frequency region followed by an inclined spike at lower frequencies, which is characteristic of ionically conducting liquid electrolytes. The semicircle corresponds to the bulk resistance (*R*_b_) and interfacial contributions, while the low-frequency tail is associated with electrode polarization and diffusion-controlled processes. The bulk resistance was obtained from the intercept of the semicircle with the real axis (*Z*′). A clear decrease in the semicircle diameter is observed with increasing DMSO content, indicating reduced bulk resistance and enhanced ionic transport. The ionic conductivity (*σ*) was calculated using:4
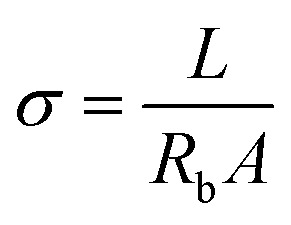
where *L* is the electrolyte thickness, *A* is the electrode–electrolyte contact area, and *R*_b_ is the bulk resistance. The conductivity increases significantly with DMSO addition, reaching a maximum value of ∼1.7 × 10^−3^ S cm^−1^ for HFE@400 µL DMSO at room temperature. This enhancement is attributed to improved salt dissociation and reduced ion pairing due to the high dielectric constant and strong coordinating ability of DMSO, which facilitates Mg^2+^ transport. Fig. 2(b) shows the Arrhenius plots of ln(*σ*) *versus* 1000/T, suggesting thermally activated ionic conduction. The conductivity follows the Arrhenius relation:^23^5
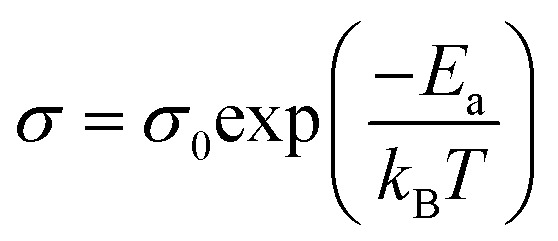
where *E*_a_ is the activation energy. The extracted values (∼0.10–0.12 eV) indicate a low energy barrier for ion migration. The HFE@400 µL DMSO electrolyte exhibits the lowest activation energy (∼0.10 eV), consistent with its highest conductivity, suggesting that DMSO enhances Mg^2+^ mobility by modifying the solvation structure. Fig. 2(c) illustrates the dielectric response in terms of the imaginary part (*ε*″) *versus* the real part (*ε*′). The behavior reflects dipolar relaxation and interfacial polarization effects. At lower frequencies, both *ε*′ and *ε*″ increase due to electrode polarization and space charge accumulation, while at higher frequencies the response stabilizes as dipoles fail to follow the rapidly alternating field. This suggests that the dielectric behavior is governed by thermally activated relaxation processes. The relaxation time (*τ*) was determined from the characteristic frequency corresponding to the maximum dielectric loss using:^24^6
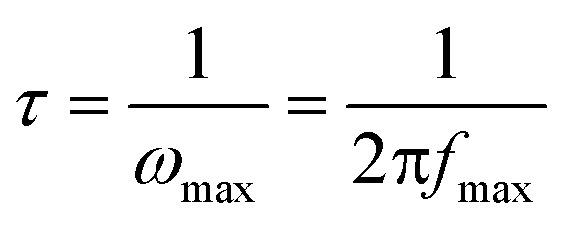
where *f*_max_ is the frequency at which the loss peak occurs. Fig. 2(d) shows the temperature dependence of *τ*. The relatively stable values of *τ* for HFE and low DMSO concentrations indicate a consistent relaxation mechanism. However, for HFE@400 µL DMSO, a slight increase in *τ* with temperature is observed, suggesting stronger Mg^2+^-DMSO coordination and a more structured solvation environment, which slightly restricts dipole reorientation despite improved ionic conduction. Fig. 2(e and f) presents the impedance and polarization behavior before and after DC polarization, used to determine the Mg^2+^ ion transference number. The transference number (*t*_Mg^2+^_) was calculated using the Bruce–Vincent–Evans equation:^25^7
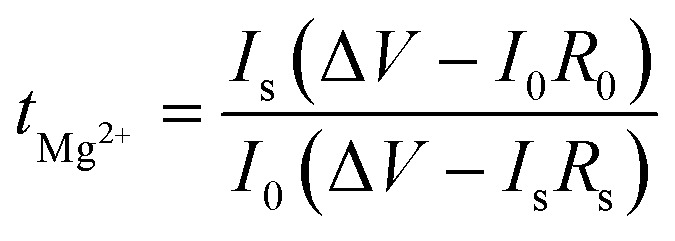
where *I*_0_ and *I*_s_are the initial and steady-state currents, *R*_0_and *R*_s_are the resistances before and after polarization, and ΔV = 0.005 V. The calculated values (Table 1) show an increase in *t*_Mg^2+^_ from 0.74 for HFE to 0.82 for HFE@400 µL DMSO, indicating that Mg^2+^ ions dominate the charge transport. The increase in steady-state current for HFE@400 µL DMSO is attributed to gradual interfacial activation during DC polarization. DMSO promotes the formation of a more stable and less resistive Mg/electrolyte interphase, enhancing Mg^2+^ transport and reducing interfacial polarization over time.^26,27^ This enhancement suggests that DMSO suppresses anion mobility and promotes selective Mg^2+^ conduction, reducing concentration polarization and improving electrochemical efficiency. Fig. 2(g) shows the linear sweep voltammetry (LSV) curves for HFE and HFE@400 µL DMSO over the voltage range −1 to 5 V. Both electrolytes exhibit low current densities at low potentials, indicating good reductive stability. The onset of oxidation occurs at ∼3.7 V for HFE and shifts to ∼2.95 V upon DMSO addition. The oxidation onset potential was determined from the voltage at which a continuous increase in anodic current density was observed in the LSV curves. The addition of DMSO significantly modifies the anodic response and electrochemical behavior of the electrolyte system, indicating altered interfacial and charge-transfer characteristics at high potentials. The LSV results indicate that DMSO significantly influences the anodic electrochemical behavior of the electrolyte, although the observed increase in anodic current at high potentials may also suggest enhanced electrolyte decomposition reactions.

**Table 1:** Electrochemical parameters (*I*_0_, *I*_s_, *R*_0_, *R*_s_) and Mg^2+^ transference number (*t*_Mg^2+^_) for HFE and HFE@400 µL DMSO

Sample	*I*_0_ µA	*I*_s_ µA	*R*_0_ Ω	*R*_s_ Ω	*t*_Mg^+2^_
HFE	1.85	1.65	51.616	70	0.7373046
HFE@ _400µL_ DMSO	16.89	7.80	25	30.5	0.8197474

**Fig. 3:**
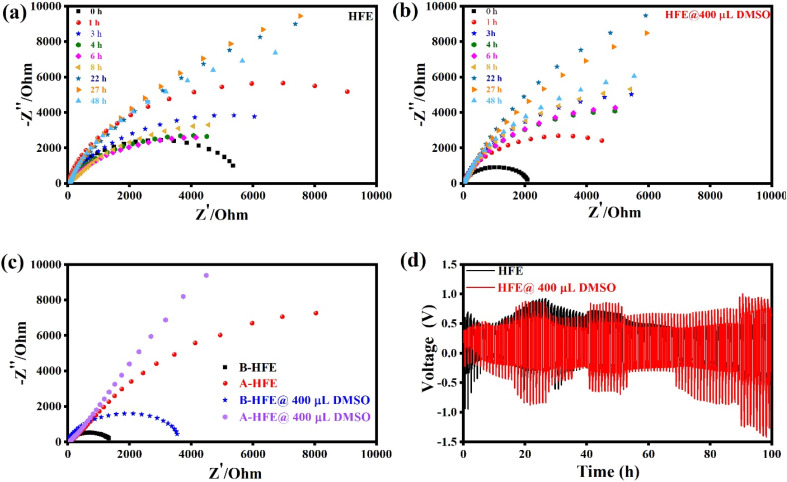
(a and b) Time-dependent Nyquist plots of HFE and HFE@400 µL DMSO. (c) Mg|Mg impedance before/after polarization. (d) Stripping/plating profiles at 20 µA cm^−2^.

Fig. 3(a and b) shows the time-dependent AC impedance (Nyquist plots) of HFE and HFE@400 µL DMSO electrolytes at different rest times. The impedance evolution with rest time suggests gradual interfacial changes at the Mg/electrolyte interface. The observed increase–decrease–increase trend in the Nyquist semicircle is interpreted as possible interfacial evolution and restructuring during resting. However, these interpretations are qualitative and mainly based on the visual evolution of the impedance spectra rather than direct evidence of specific interphase formation or dissolution mechanisms.^28^ For both systems, an initial increase in the semicircle diameter (interfacial resistance) is observed from 0 to 1 h, which is attributed to the formation of an interfacial layer at the Mg surface resulting from electrolyte decomposition and Mg/electrolyte reactions.^26^ This layer partially blocks Mg^2+^ transport, leading to increased interfacial resistance. At intermediate rest times (3–6 h), a decrease in resistance is observed, suggesting possible interfacial restructuring or partial stabilization. However, the resistance increases again at longer rest times (8–27 h), indicating continued interfacial evolution during resting. Compared with pristine HFE, the HFE@400 µL DMSO electrolyte exhibits lower overall impedance and slower resistance growth, suggesting that DMSO influences the Mg/electrolyte interfacial behavior. Fig. 3(c) further shows that the HFE@400 µL DMSO system exhibits a smaller semicircle than HFE, indicating reduced interfacial resistance and improved Mg^2+^ transport kinetics, consistent with the higher ionic conductivity and Mg^2+^ transference number observed for the DMSO-containing electrolyte. Although both Fig. 2(e) and Fig. 3(c) involve impedance measurements before and after polarization, they were used for different electrochemical analyses. Fig. 2(e) was primarily employed for Mg^2+^ transference number determination through DC polarization measurements, whereas Fig. 3(c) was used to evaluate the evolution of Mg/electrolyte interfacial behavior and impedance stability after polarization. Fig. 3(d) shows the galvanostatic Mg stripping/plating profiles for HFE and HFE@400 µL DMSO at a current density of 20 µA cm^−2^. The relatively low current density (20 µA cm^−2^) was employed to minimize polarization effects and accurately evaluate the intrinsic Mg stripping/plating behavior and interfacial stability of the developed halogen-free electrolyte. Such low current densities are commonly used in Mg battery studies due to the sluggish Mg^2+^ transport kinetics and strong solvation effects, which can cause significant overpotential and interfacial instability at higher current densities^29,30^. The overpotential values were compared based on the average voltage hysteresis observed during repeated Mg stripping/plating cycles under identical current density conditions. The areal capacity per half-cycle was approximately ∼0.018 mAh cm^−2^. In addition, the Mg‖Mg measurements were repeated using independently assembled symmetric cells, showing acceptable reproducibility under identical experimental conditions. The HFE electrolyte exhibits relatively high and unstable overpotential, particularly at the initial stages, due to the formation of a resistive passivation layer on the Mg surface. In contrast, the HFE@400 µL DMSO electrolyte demonstrates significantly lower and more stable overpotential (below ∼1 V) over extended cycling (∼90 h). The reduced polarization indicates a lower energy barrier for Mg^2+^ transport and more efficient charge-transfer processes at the interface. Moreover, stable voltage hysteresis over long cycling time suggests that the addition of DMSO effectively suppresses continuous interfacial degradation and promotes the formation of a more stable and ion-conductive interphase. This enhanced interfacial stability directly contributes to improved cycling performance and suggests that DMSO plays a critical role in enabling reversible Mg stripping/plating behavior.

**Fig. 4:**
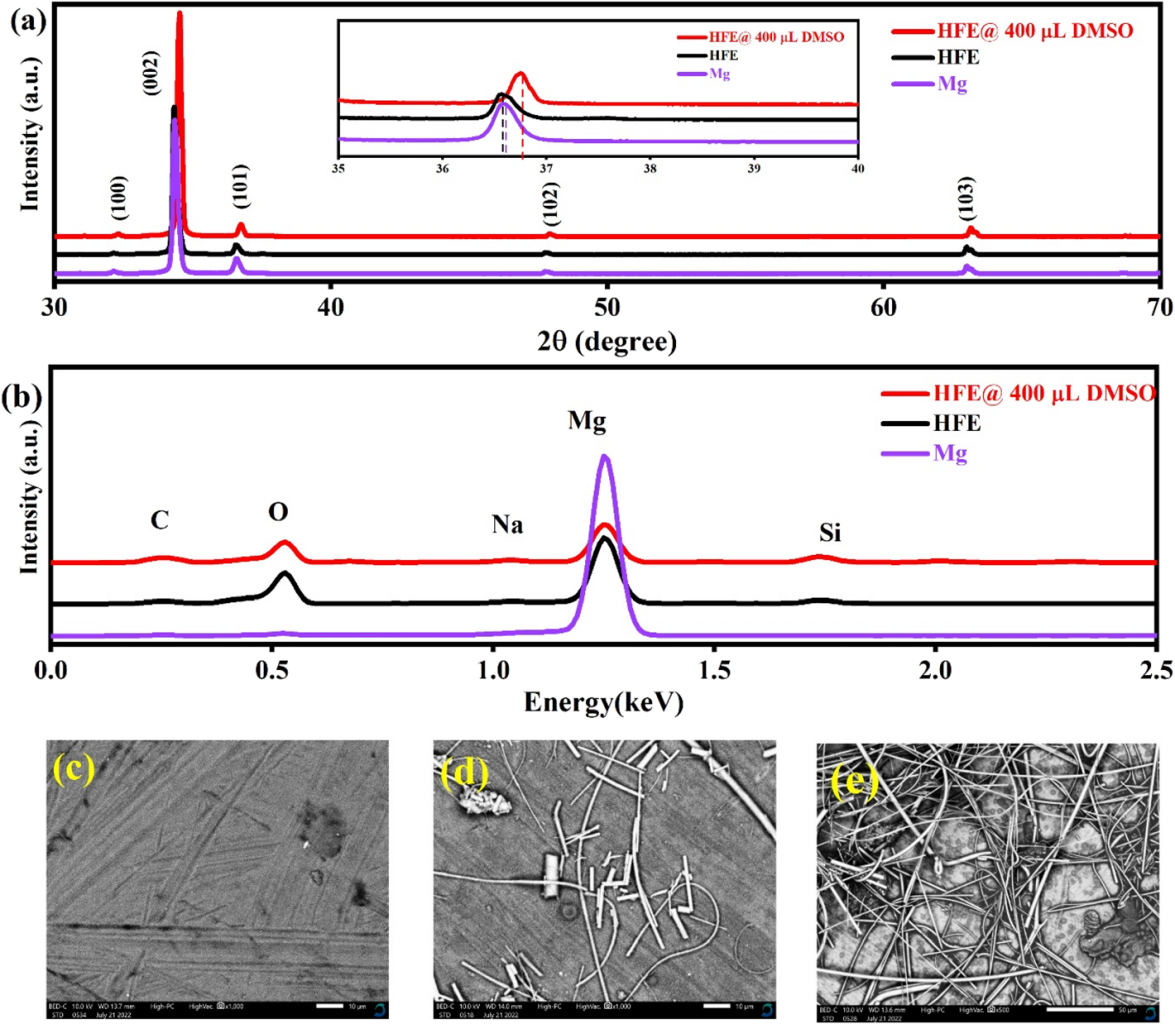
(a) XRD patterns of Mg, Mg_HFE, and Mg_HFE@400 µL DMSO. (b) EDS spectra. (c–e) SEM images showing surface morphology.

**Fig. 5:**
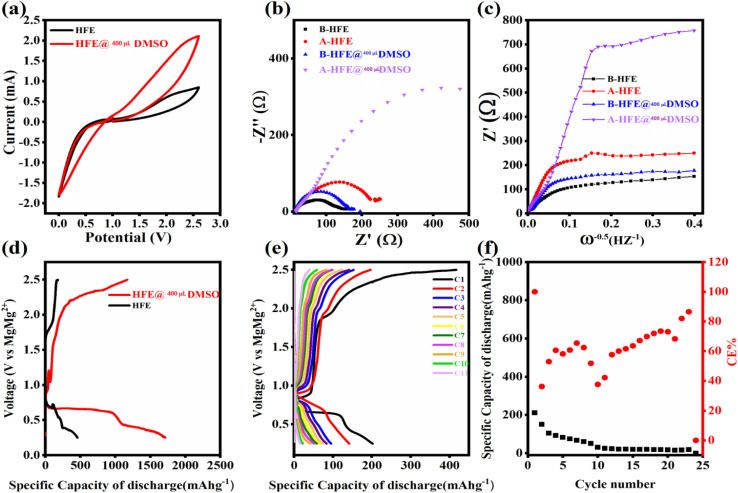
(a) CV of full cells for HFE and HFE@400 µL DMSO. (b) EIS before/after CV. (c) *Z*′ *vs. ω*^−0.5^ (d) first charge–discharge profiles. (e) Cycling curves of HFE@400 µL DMSO after CV (f) specific capacity and coulombic efficiency *vs.* cycle number.

Fig. 4(a) shows the X-ray diffraction (XRD) patterns of pristine Mg, Mg after contact with HFE, and Mg after contact with HFE@400 µL DMSO. The diffraction peaks located at 2*θ* ≈ 32.1°, 34.3°, 36.5°, 47.7°, 57.2°, and 62.9° are indexed to the (100), (002), (101), (102), (110), and (103) planes, respectively, corresponding to the hexagonal close-packed (hcp) structure of magnesium (JCPDS No. 901-3058).^31^ For the Mg_HFE sample, a slight shift of the diffraction peaks toward lower angles is observed, indicating an increase in lattice spacing (*d*-spacing), which can be attributed to surface strain or the formation of a loosely bound interfacial layer due to electrolyte decomposition. In contrast, the Mg_HFE@400 µL DMSO sample exhibits a small shift toward higher angles, suggesting lattice contraction or the formation of a more compact and stable interfacial layer. Additionally, a reduction in peak intensity is observed after electrolyte exposure, which indicates partial surface coverage and decreased crystallinity due to the formation of an interphase layer. The shift in peak position can be interpreted using Bragg's law:

8 *nλ* = 2*d*sin *θ*

where *d* is the interplanar spacing and *θ* is the diffraction angle. A shift toward lower angles corresponds to an increase in *d*, while a shift toward higher angles indicates a decrease in *d*, suggesting structural modification of the Mg surface upon electrolyte interaction. Fig. 4(b) presents the EDS spectra of the Mg surface after interaction with the electrolytes. In addition to the dominant Mg peak, new signals corresponding to C and Na are observed for the Mg_HFE sample, originating from the electrolyte components. For the Mg_HFE@400 µL DMSO sample, an additional Si signal is detected, which may arise from residual separator. The observed surface morphology changes after electrolyte exposure suggest modification of the Mg/electrolyte interface. However, the SEM results alone do not directly confirm the formation of a more ion-conductive interphase. The weak Na signal detected in the EDS analysis may originate from trace contamination, residual impurities, or residual separator species during sample handling and post-cycling analysis. Therefore, the XRD, EDS, and SEM results are interpreted as evidence of interfacial evolution and surface chemistry modification after electrolyte exposure rather than direct confirmation of specific interphase composition or conductivity. Fig. 4(c–e) shows SEM micrographs of pristine Mg and Mg after exposure to the electrolytes. The pristine Mg surface appears relatively smooth and uniform, indicating a clean metallic surface. After contact with HFE, the surface becomes rough and irregular, suggesting the formation of a thin passivation layer. In contrast, the Mg_HFE@400 µL DMSO sample exhibits a more structured and uniformly distributed surface morphology, characterized by interconnected features that indicate the formation of a more stable and compact interphase layer. This morphological evolution suggests that DMSO promotes the formation of a controlled and ion-conductive interfacial layer, which is consistent with the improved electrochemical performance observed in impedance and cycling measurements. Overall, the combined XRD, EDS, and SEM analyses suggest that the addition of DMSO significantly alters the surface chemistry and morphology of Mg, leading to the formation of a more stable and less resistive interphase. This stabilized interface plays a crucial role in enhancing Mg^2+^ transport and suppressing continuous passivation, thereby improving the overall performance of the electrolyte system. Fig. 5(a) shows the cyclic voltammetry (CV) curves of HFE and HFE@400 µL DMSO electrolytes measured between 0.3 and 2.5 V at a scan rate of 0.008 V s^−1^. Both systems exhibit broad cathodic and anodic features corresponding to Mg^2+^ insertion/extraction processes. The oxidation peaks are relatively weak and broadened, reflecting the sluggish kinetics of Mg^2+^ due to its divalent nature and strong solvation. The anodic peak is located around ∼2.02 V for HFE and shifts to ∼2.44 V for HFE@400 µL DMSO, indicating improved electrochemical activity and extended anodic stability upon DMSO addition. Furthermore, the higher current response observed for HFE@400 µL DMSO suggests enhanced charge-transfer kinetics and improved reversibility. Fig. 5(b and c) presents the electrochemical impedance spectra before and after CV measurements, along with the corresponding Warburg plots (*Z*′ *vs. ω*^−0.5^). The spectra were fitted using an equivalent circuit consisting of solution resistance (*R*_s_), interfacial resistance (*R*_SEI_), charge-transfer resistance (*R*_ct_), and Warburg impedance. The real part of the impedance in the low-frequency region follows:^32^

**Fig. 6:**
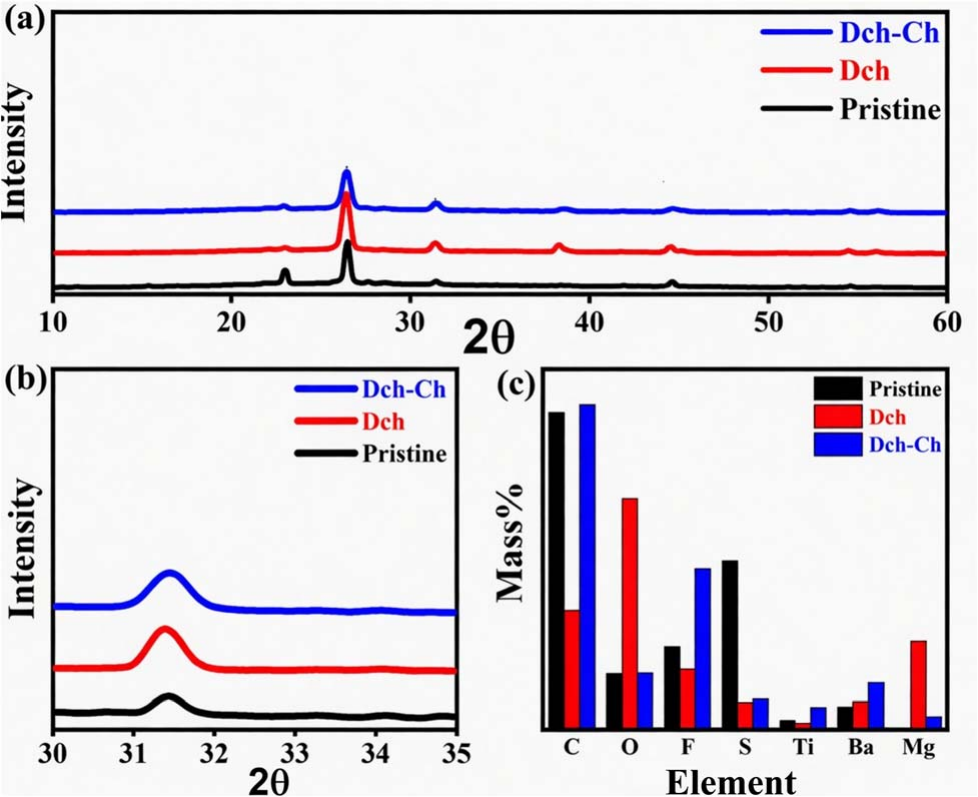
(a) XRD patterns of the cathode (pristine, Dch, Dch–Ch). (b) Enlarged region. (c) Elemental composition.

9 *Z*′ = *R*_s_ + *R*_ct_ + *σω*^−1/2^

where *σ* (often denoted as *A*_w_) is the Warburg coefficient. The parameters extracted using ZView are summarized in Table 2. The magnesium diffusion coefficient (*D*_Mg^2+^_) was calculated using:^32^10
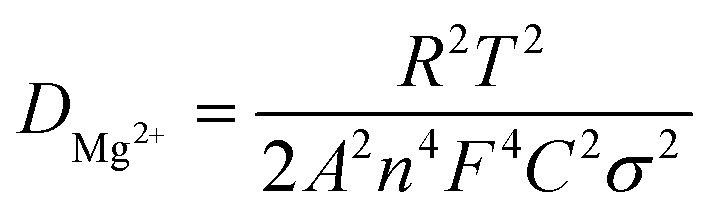
where *R* is the gas constant (8.314 J mol^−1^ K^−1^), *T* is the absolute temperature (298 K), suggesting that DMSO facilitates ion transport by modifying the solvation structure and reducing diffusion limitations, *A* is the electrode area (1.5 cm^2^), *n* = 2 is the number of transferred electrons, *F* is Faraday's constant (96 485 C mol^−1^), *C* is the Mg^2+^ concentration (4 × 10^−4^ mol cm^−3^), and *σ* is the Warburg coefficient. The calculated values show that *D*_Mg^2+^_ increases from 1.24 × 10^−13^ cm^2^ s^−1^ for HFE to 2.47 × 10^−12^ cm^2^ s^−1^ for HFE@400 µL DMSO, indicating significantly enhanced Mg^2+^ diffusion in the presence of DMSO. The significant enhancement in the Mg^2+^ diffusion coefficient after DMSO addition is attributed to the high dielectric constant and strong coordinating ability of DMSO, which weaken Mg^2+^–anion interactions and suppress ion pairing. This promotes a more dissociated solvation structure, facilitating faster Mg^2+^ transport and lowering diffusion resistance at the electrode/electrolyte interface. Consequently, DMSO improves charge-transfer kinetics and reduces the Warburg impedance of the electrolyte system.^33^ This improvement is consistent with the reduced charge-transfer resistance (*R*_ct_) and lower Warburg coefficient, suggesting that DMSO facilitates ion transport by modifying the solvation structure and reducing diffusion limitations. Fig. 5(d) shows the first galvanostatic charge–discharge profiles at a current density of 20 µA cm^−2^. The HFE electrolyte exhibits relatively low discharge/charge capacities (∼471/180 mAh g^−1^), whereas HFE@400 µL DMSO shows significantly higher capacities (∼1732/1176 mAh g^−1^). The theoretical specific capacity of MgS is 1675 mAh g^−1^. The experimentally observed first-cycle discharge capacity (1732 mAh g^−1^) slightly exceeds the theoretical capacity of Mg–S batteries. This behavior is mainly attributed to irreversible electrochemical processes during the initial discharge, including electrolyte decomposition, SEI formation, interfacial side reactions, and surface-controlled charge storage. Moreover, the highly polar aprotic nature of DMSO may promote parasitic reactions, leading to an apparent increase in the initial discharge capacity.^34^ The voltage plateaus observed in the ranges of ∼1.0–2.2 V (charge) and ∼0.58–0.67 V (discharge) correspond to the redox conversion reactions of sulfur species. The enhanced capacity in the DMSO-containing electrolyte is attributed to improved Mg^2+^ transport, better electrode/electrolyte interface, and enhanced utilization of active material. Fig. 5(e) presents the charge–discharge profiles over multiple cycles (after initial conditioning). A gradual decrease in capacity with cycling is observed, with initial values of ∼418/202 mAh g^−1^. This capacity fading is attributed to incomplete reversibility of Mg^2+^ insertion/extraction, formation of passivation layers, and possible trapping of Mg^2+^ ions within the sulfur matrix. Additionally, polysulfide dissolution and shuttling effects contribute to the loss of active material and reduced capacity retention. Fig. 5(f) shows the cycling performance and coulombic efficiency. The discharge capacity initially decreases, followed by a gradual increase with cycling, indicating an activation process of the electrode/electrolyte interface. The coulombic efficiency initially fluctuates but subsequently increases, reaching ∼90% at cycle 23. The cycling test was limited to 25 cycles due to the pronounced capacity fading at extended cycling, which is mainly attributed to the gradual growth of passivation layers on the Mg anode, leading to increased interfacial resistance and hindered reversible Mg^2+^ transport, in addition to polysulfide-related side reactions. This improvement suggests enhanced reversibility and stabilization of the electrochemical system upon cycling. The gradual increase in coulombic efficiency during the initial cycles can be attributed to an activation process occurring at the electrode/electrolyte interface.^3^ During the early cycling stages, partial electrolyte decomposition and interphase formation consume charge and reduce the coulombic efficiency.^35^ Upon continued cycling, a more stable and ion-conductive interfacial layer is formed, which suppresses side reactions and improves the reversibility of Mg^2+^ insertion/extraction processes.^36^ This stabilization leads to the observed increase in coulombic efficiency to approximately 90% after several cycles. The overall behavior suggests that the addition of DMSO improves Mg^2+^ transport, reduces polarization, and enhances electrochemical performance, although long-term stability remains influenced by interfacial reactions and polysulfide-related effects. Fig. 6(a–c) presents the XRD patterns of the cathode in the pristine, discharged (Dch), and discharged–charged (Dch–Ch) states. In the pristine sample, the diffraction pattern exhibits characteristic peaks corresponding to crystalline sulfur,^37^ along with a broad feature around ∼24–26° associated with the (002) plane of graphitic carbon. The sulfur peaks serve as a reference to monitor structural evolution during electrochemical cycling. Upon discharge, noticeable changes occur in the sulfur-related peaks. Specifically, a reduction in peak intensity and slight broadening are observed, indicating the consumption of crystalline sulfur and the formation of less-ordered or amorphous discharge products. This behavior is consistent with the conversion of sulfur into intermediate magnesium polysulfide species and/or Mg-containing compounds, which are typically poorly crystalline and therefore not clearly resolved in XRD patterns. After the subsequent charging process (Dch–Ch), a partial recovery of the sulfur peak intensity is observed, indicating the reformation of crystalline sulfur from the discharge products. However, the restored peaks remain broader and less intense compared to the pristine state, suggesting incomplete reversibility of the conversion reaction. This incomplete recovery can be attributed to residual amorphous species, Mg^2+^ trapping within the cathode matrix, and irreversible side reactions. The magnified region in Fig. 6(b) further suggests these trends, showing peak broadening and slight positional variations, which reflect structural disorder and lattice distortion induced by repeated Mg^2+^ insertion/extraction. Importantly, no well-defined new crystalline phases are clearly detected, supporting the conclusion that the electrochemical reaction proceeds predominantly through amorphous or poorly crystalline intermediates. Fig. 6(c) shows the elemental composition (mass%) of the cathode in different states. In the discharged state, an increase in Mg content is observed alongside a relative decrease in sulfur content, suggesting the insertion of Mg^2+^ and the consumption of sulfur during discharge. Upon charging, the Mg content decreases while the sulfur content increases again, indicating partial reversibility of the reaction. However, the sulfur content does not fully return to its original level, which supports the XRD observation of incomplete structural recovery. Overall, the combined XRD and compositional analysis demonstrate that the electrochemical process involves a conversion reaction of sulfur with Mg^2+^, characterized by the transformation of crystalline sulfur into amorphous discharge products and its partial regeneration upon charging. The evolution of sulfur peaks provides direct evidence of this reversible, yet partially irreversible, Mg–S reaction mechanism.

**Table 2:** EIS-derived parameters (*R*_s_, *R*_SEI, *R*_ct, Warburg coefficient) and Mg^2+^ diffusion coefficient (*D*_Mg^2+^_) for HFE and HFE@400 µL DMSO

EIS parameter	*R*_s_ Ω	*R*_SEI_ Ω	*R*_ct_ Ω	*A*_w_ Ω	*D*_Mg^2+^_ [cm^2^ s^−1^]
B-HFE	12.78	46.47	349.8	144.48811	1.24 × 10^−13^
B-HFE@_400µL_DMSO	14.44	60.153	279.2	32.40478	2.47 × 10^−12^

**Fig. 7:**
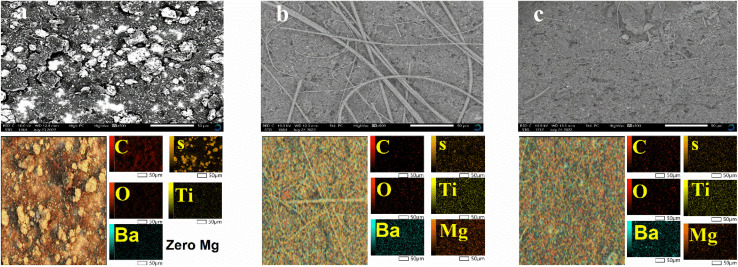
SEM images and EDS mapping of the cathode in (a) pristine, (b) discharged (Dch), and (c) discharged–charged (Dch–Ch) states.

Fig. 7(a–c) presents SEM micrographs and corresponding EDS elemental mapping of the cathode in the pristine, discharged (Dch), and discharged–charged (Dch–Ch) states. In the pristine state (Fig. 7(a)), the cathode exhibits a heterogeneous granular morphology with well-distributed elements (C, O, S, Ti, and Ba) and no detectable Mg signal, suggesting the absence of Mg^2+^ prior to electrochemical operation. After discharge (Fig. 7(b)), the surface morphology becomes more covered and uniform, indicating the formation of a reaction layer associated with Mg^2+^ insertion and conversion reactions. The EDS mapping clearly shows the appearance and homogeneous distribution of Mg across the cathode surface, suggesting the incorporation of Mg^2+^ ions into the electrode. This change is consistent with the formation of Mg-containing discharge products within the sulfur-carbon matrix. Upon subsequent charging (Fig. 7(c)), the surface appears relatively smoother with reduced coverage compared to the discharged state, suggesting partial removal or redistribution of the discharge products. The Mg signal remains present but at a lower intensity, indicating incomplete extraction of Mg^2+^ ions from the cathode. Overall, the SEM and EDS analyses suggest that Mg^2+^ ions are inserted into the cathode during discharge and partially extracted upon charging, accompanied by morphological evolution of the electrode surface. The persistence of Mg after charging indicates partial irreversibility, which is consistent with Mg^2+^ trapping and contributes to capacity fading during cycling.

## Conclusion

In this study, dimethyl sulfoxide (DMSO) was successfully employed as a functional additive to enhance the performance of a halogen-free Mg(NO_3_)_2_-based electrolyte for magnesium battery applications. The incorporation of DMSO significantly modified the solvation structure through strong coordination with Mg^2+^ ions, resulting in improved ionic conductivity (1.7 × 10^−3^ S cm^−1^), reduced activation energy, and an increased Mg^2+^ transference number (∼0.82). Electrochemical measurements suggested enhanced anodic stability, reduced overpotential, and improved Mg stripping/plating behavior, indicating a stabilized Mg/electrolyte interface. Furthermore, the integration of a sulfur/graphene/BaTiO_3_ composite cathode enabled high initial discharge/charge capacities and improved electrochemical performance in full-cell configurations. Structural and morphological analyses revealed that DMSO promotes the formation of a more uniform and less resistive interphase on the Mg surface, while also facilitating Mg^2+^ transport within the cathode. Despite these improvements, partial irreversibility associated with Mg^2+^ trapping and polysulfide-related processes still contributes to capacity fading during cycling. Overall, this work establishes a clear structure–property–performance relationship for DMSO-modified halogen-free electrolytes and highlights solvation-structure engineering as a viable pathway toward the development of high-performance, non-corrosive magnesium battery systems.

## Conflicts of interest

There are no conflicts to declare.

## Data Availability

The data supporting the findings of this study are available within the article. Additional data related to this work are available from the corresponding author upon reasonable request.
